# Test-fairness deep learning with influence score

**DOI:** 10.1371/journal.pdig.0001513

**Published:** 2026-07-16

**Authors:** Jacky Chung-Hao Wu, Chang-Yu Shih, Nien-Chen Wu, Wei-Wen Chen, Henry Horng-Shing Lu, Shaw-Hwa Lo

**Affiliations:** 1 Center for Fundamental Science, Kaohsiung Medical University, Kaohsiung, Taiwan; 2 Biomedical Artificial Intelligence Academy, Kaohsiung Medical University, Kaohsiung, Taiwan; 3 Institute of Statistics, National Yang Ming Chiao Tung University, Hsinchu, Taiwan; 4 Institute of Data Science and Engineering, National Yang Ming Chiao Tung University, Hsinchu, Taiwan; 5 Institute of Artificial Intelligence Innovation, National Yang Ming Chiao Tung University, Hsinchu, Taiwan; 6 Institute of Computer Science and Engineering, National Yang Ming Chiao Tung University, Hsinchu, Taiwan; 7 Department of Artificial Intelligence in Medicine, Kaohsiung Medical University, Kaohsiung, Taiwan; 8 Department of Medical Research, Kaohsiung Medical University Hospital, Kaohsiung, Taiwan; 9 Department of Statistics and Data Science, Cornell University, Ithaca, New York, United States of America; 10 Department of Statistics, Columbia University, New York, New York, United States of America; University of Washington, UNITED STATES OF AMERICA

## Abstract

Performance disparities in AI systems can manifest across sensitive groups or across data sources, especially when training data are collected from specific populations. In this work, we propose a feature-selection-based method that improves test-fairness while preserving prediction performance. Built on deep learning models, the proposed approach adopts the influence score (I-score), a statistical measure that captures interaction effects among multiple features. We identify features strongly associated with dataset membership by training an auxiliary model to predict dataset origin and applying I-score-based subset selection; these dataset-associated features are then excluded (masked) from the original prediction model for follow-up inference. We conduct experiments on two skin lesion datasets, ISIC 2019 and ASAN, collected from different populations. The empirical results show that the resulting fair I-score model can maintain high classification performance for skin lesion prediction while reducing cross-dataset subgroup performance disparity under our test-fairness evaluation setting.

## Author summary

Artificial intelligence is increasingly used to support medical image interpretation, but models that look accurate overall can still perform differently across patient populations or healthcare settings. In practice, medical image datasets often come from specific hospitals or regions, and images may differ in camera type, lighting, workflow, and population characteristics. As a result, a model may learn patterns that are tied to the data source rather than to clinically meaningful signs of disease, which can lead to unequal performance when the model is applied elsewhere. In this study, we present a practical framework to evaluate and reduce such uneven behavior without collecting new data or changing the original datasets. Using skin lesion classification as an example, we identify internal model signals that are strongly linked to the dataset source and reduce the model’s reliance on them. We test the approach on two widely used datasets from different populations (ASAN and ISIC 2019) and assess external generalization on a third dataset (PAD-UFES-20). The results show that the revised model maintains strong diagnostic performance while reducing cross-dataset performance disparity under our test-fairness evaluation setting, supporting more reliable deployment across clinical contexts.

## 1 Introduction

The development of artificial intelligence is becoming more and more mature, and people can solve various problems to a great extent through artificial intelligence, such as image recognition, language translation, and classification problems. Most deep learning models are used as black boxes, but as people rely more and more on artificial intelligence, how to ensure the fairness of the prediction results of a model has also become an important issue [1]. This study focuses on fairness in medical AI and how it can be evaluated and improved in practice.

To frame this work, fairness in machine learning can be defined in multiple ways depending on the task and the sensitive attribute of interest. Common formulations include reducing subgroup performance disparities (e.g., differences in sensitivity or specificity), constraining error rates across groups (e.g., equalized odds/equal opportunity), and calibration-based criteria. In this work, we focus on test-fairness as subgroup performance parity under a fixed evaluation setting, and we quantify unfairness using sensitivity differences (see Section 2.4.4 for a detailed discussion of fairness definitions).

In practice, fairness in AI is often discussed in terms of whether a model yields comparable predictive performance across sensitive groups (e.g., defined by gender, sexual orientation, or race) and does not systematically benefit or disadvantage specific populations [2–4]. Taking the problem of skin lesion classification as an example, the model should obtain similar accuracy when faced with different gender, region, and other sensitive data without favoring a specific ethnic group. The distribution of the data is a significant factor that causes the model to have unfair behavior. Suppose the collection of the data is concentrated in a specific group or population. In that case, the chance of the model learning the characteristics of the particular group during training will greatly increase, potentially leading to reduced generalization and subgroup performance disparities for other groups. The unfair model also occurs frequently in healthcare because most patients’ data will not be shared between hospitals due to privacy issues. So most of the collected patient data come from the same hospitals, and the dataset will have more or fewer deviations, so the issue of fairness is also a big challenge in healthcare.

**Fig 1:**
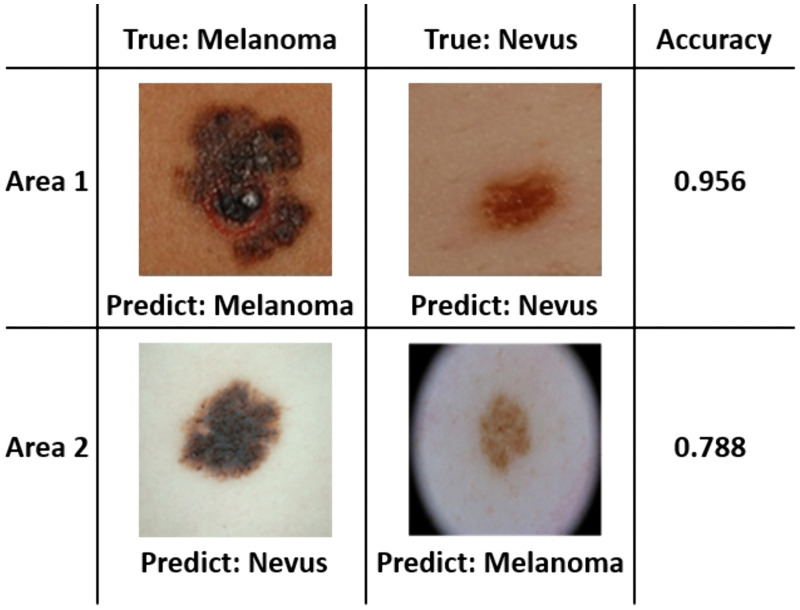
Cross-dataset performance disparity in skin lesion classification. Area 1 (ASAN) and Area 2 (ISIC 2019) are collected from different populations. Cross-dataset performance gaps may reflect distributional shift and/or potential subgroup-related unfairness, which is quantified in this study using test-fairness based on subgroup sensitivity differences.

Fig 1 illustrates a cross-dataset performance disparity observed when transferring a VGG16-based skin lesion classifier [5]. The model is trained on the ASAN dataset (Area 1; collected primarily in South Korea) and evaluated on both ASAN and the ISIC 2019 dataset (Area 2), a widely used public benchmark for skin lesion classification. The prediction performance on Area 2 is substantially lower than that on Area 1. We note that such cross-dataset performance gaps can arise from multiple factors, including distributional shift between data sources, and should not be interpreted as definitive evidence of subgroup-related bias in isolation. Accordingly, we quantify unfairness in this work using the test-fairness definition (sensitivity differences across sensitive groups under a fixed evaluation setting), which provides a direct measure of subgroup performance disparity.

To mitigate subgroup performance disparity of the model and improve the fairness of the model, it can start with the pre-processing of the data or the model itself. When pre-processing the data, we add some restrictions to make the population distribution of the dataset as even as possible so that the model is less likely to learn dataset-associated features during training. Although dealing with data distribution can reduce the unfair behavior of the model, it is difficult to achieve in practice due to data collection problems. Besides, different pre-processing strategies may need to be applied for various issues, which is not always feasible. Therefore, this study focuses on the model and proposes the fair I-score method to improve the fairness of the model. The key point of this method is to find out the dataset-associated features encoding the sensitive information of some specific groups in the feature set learned by the model and improve fairness while preserving predictive accuracy by eliminating dataset-associated features.

Recent years have seen a rapidly growing interest in fairness for deep learning-based medical image analysis, with systematic reviews documenting a substantial body of work on fairness evaluation and mitigation across imaging modalities and clinical tasks [6]. Prior studies have reported clinically meaningful performance disparities associated with demographic or site-related factors, often driven by dataset composition, population heterogeneity, and acquisition protocols [7,8]. These observations motivate the need for methods that can both diagnose sources of unfair behavior and mitigate them in a way that is compatible with existing CNN pipelines. In this context, our work contributes a feature-level, interaction-aware perspective based on the influence score (I-score) to identify and remove dataset-associated features while preserving diagnostic performance.

In addition to fairness-specific research, related challenges such as distributional shift and predictive drift have been extensively studied in broader machine learning contexts. Prior work in areas including social media analytics and medical image analysis has shown that changes in data distributions across time, populations, or data sources can substantially affect model reliability and generalization performance. These studies highlight that performance disparities may arise not only from explicit model bias, but also from distributional differences between training and deployment settings. Such observations provide a broader context for fairness evaluation, as distributional heterogeneity across populations is often a key factor underlying unequal predictive performance (e.g., [9,10]).

In 2009 and 2016, Lo *et al.* [11,12] proposed the concept of the influence score, I-score. In statistics, the I-score indicates that not every feature may necessarily be important for making predictions. The most critical aspect of the I-score is to determine the subset of features with interactions that jointly influence the class labels. In [12], Lo *et al.* demonstrated how subsets of features are selected using the I-score. I-score values indicate the importance of each subset of features; the more critical a subset of features is, the higher the value is. The method of effectively selecting influential feature subsets from data with the backward dropping algorithm [11] has been employed in several analyses of numerical data and simulations [13]. I-score plays an essential role in our proposed fair I-score method and helps us obtain the dataset-associated features from the global average pooling (GAP) layer of the CNN model. By eliminating the dataset-associated features, we can build the fair I-score model using only fair features.

Moreover, the visualization method, Grad-CAM [14], is adopted to demonstrate the effectiveness of the proposed I-score method. In 2016, Zhou *et al.* [15] proposed the class activation mapping (CAM) to explain the essential characteristics of regions in the image emphasized by convolutional neural networks (CNNs) for determining the classification of the entire image. CAMs use the values of images in the GAP layer of a CNN model to highlight the regions in the original images that contribute to the classification of one specific class. Furthermore, a CAM visualizes the weights of each category feature map in the form of a heat map. However, CAM can only apply to the model with the GAP layer followed by a single output layer. To reserve the extensibility of our proposed fair I-score method to more complicated model architectures, we use Grad-CAM to visualize most of the prediction models generally. The main difference between CAM and Grad-CAM is the way to get the weights. Because most network architectures are nonlinear, Grad-CAM approximates the class score as a locally linear function of the last convolutional feature maps. Specifically, Grad-CAM uses the gradients of the target class score with respect to each feature map to compute an importance weight, which corresponds to a first-order (linear) approximation of how changes in that feature map affect the class score. This linear approximation is important because it produces a class-discriminative and spatially resolved heatmap that remains directly linked to the model’s decision while being applicable to a broad range of CNN architectures. After generating the weights, the weighted feature maps will be plotted on the images to visualize the main features.

In this work, we focus on CNN-based image classifiers with a GAP layer, and we use a VGG16-based architecture as a concrete example. We modify the original architecture by replacing the flatten layer with a GAP layer, which has been shown to provide better generalization performance. The fair I-score method introduces a unique approach to mitigate subgroup performance disparities in deep learning models by combining I-score-based feature selection with fairness-aware model refinement. The key novel contributions of our method are as follows:

- Feature Selection for Fairness: Unlike existing fairness techniques that often rely on adversarial training or reweighting strategies, our method leverages the I-score, a statistical measure that captures the predictive interaction strength of feature subsets. By applying this to a model trained to classify dataset origin, we systematically identify and remove dataset-associated features.
- Backward Dropping Algorithm (BDA) for Dataset-Associated Feature Removal: Our approach iteratively eliminates dataset-associated features using an I-score-driven backward selection process. This ensures that the model retains only fair, high-predictive features, improving fairness without compromising classification accuracy.
- AUC-Based Thresholding for Dataset-associated Feature Identification: We introduce a quantitative thresholding mechanism based on the maximum observed ROC AUC from the dataset-membership prediction task, enabling objective identification of features strongly associated with dataset membership while minimizing information loss.
- Generalizable Framework for Fairness Enhancement: Unlike fairness methods tailored to specific tasks, our architecture-agnostic framework can be applied to any CNN-based model with a global average pooling (GAP) layer, making it widely applicable across various domains, including medical AI, facial recognition, and NLP.

The rest is organized as follows. Section 2 introduces the concept of the I-score and the corresponding feature selection algorithm and then describes the procedure of the proposed fair I-score method. Section 3 verifies the effectiveness of the proposed fair I-score method by showing the experimental results of the proposed fair I-score method applied to the problem of skin lesion classification. Then, we discuss and conclude in Section 4.

## 2 Materials and methods

There are various definitions of fairness in AI in recent years [16]. Considering that a convolutional neural network (CNN) for classification usually produces the predicted probabilities for the outcomes, a natural choice of the definition of fairness is one that bases on the predicted probabilities and the actual outcome. Test-fairness is one kind of measure criteria for fairness based on predicted probabilities and the actual outcome [17]. A binary classifier satisfies the test-fairness if subjects with different sensitive attributes have the same probability of belonging to the positive class when the subjects have the same predicted probability score. Concretely, the definition of test-fairness is


$$\begin{array}{c} P(y=+1|s,a)=P(y=+1|s,a^{\prime }),\forall s,a,a^{\prime }, \end{array}$$ (1)

where *y* is the actual outcome. *s* denotes the predicted probability score, which is the predicted probability of the positive (or negative) class for a subject produced by the classifier. *a* and $a^{\prime }$ represent the sensitive attributes of the subjects. Similar definitions include the well-calibration [17], the balance for positive class [18], and the balance for negative class [18]. However, all the above definitions require to access the true probability distribution behind the task of interest; it is not realistic, so we turn to an operational definition of fairness which is more practical than the above definitions and will be used to evaluate the fairness of the proposed method in this study.

In the following, we will introduce the calculation of the influence score, I-score, of a subset of features and the feature selection algorithm using the calculation of the I-score, the backward dropping algorithm (BDA). Then, we describe the proposed procedure that produces the fair model. At last, we discuss the evaluation metrics of the proposed fair model for prediction performance, fairness, and visualization. We defer the introduction of the operational definition of fairness to Section 2.4. The fairness of the proposed fair model will be improved by eliminating the features governed by the sensitive attributes without sacrificing the prediction performance.

### 2.1 Influence score (I-score)

The basic idea of the influence score, I-score, is to calculate the interaction-level prediction capability of a subset of features to the target outcome. The calculation of the I-score requires all features to be discrete. If some features are continuous, they need to be converted into discrete ones for the purpose of feature selection first. After selecting the important features, the original values will be used for follow-up steps. In this study, the median is adopted as the threshold to binarize the value of continuous features. Although some information will be lost when converting the features from continuous into discrete, Wang *et al.* [13] have shown that the benefit from robust detection of feature interaction is more than enough to compensate for the information loss due to discretization.

The I-score was selected in this study because it explicitly captures interaction effects among feature subsets rather than relying solely on marginal feature importance. Unlike dependency-based measures that primarily quantify individual associations between features and the target variable, the I-score evaluates the joint contribution of feature combinations in a model-agnostic manner. While alternative interaction-based approaches exist, many require exhaustive enumeration of feature subsets or depend on model-specific assumptions, which limit their practicality for post hoc fairness assessment in high-dimensional settings. In contrast, the I-score provides an efficient and generalizable mechanism for identifying interaction patterns associated with sensitive attributes, making it well-suited for the proposed fairness evaluation framework.

In this study, we consider the task of binary classification to verify our proposed method. Furthermore, we consider the validation set of *n* examples of the *p*-dimensional binarized feature vector, $\left(x_{1},x_{2},...,x_{p}\right)$, and the associated true binary outcome *y*. Let 𝒮 be a random subset of features; without loss of generality, we assume $𝒮=\{x_{1},x_{2},...,x_{m}\}$ of size *m*. The validation set *n* examples can be divided into $2^{m}$ possible example subsets whose partition is based on the binarized values of $x_{i}$’s of 𝒮. For example, if *m* is 2, $𝒮=\{x_{1},x_{2}\}$ and there are 2^2^ = 4 possible example subsets according to $\left(x_{1},x_{2})\in \{(0,0),(0,1),(1,0),(1,1)\right\}$. The I-score of the feature subset 𝒮 is based on the corresponding partition and defined as follows,


$$\begin{array}{c} I(𝒮)=\frac{1}{n\sigma_{n}^{2}}\sum_{j=1}^{2^{m}}n_{j}^{2}{\left({\overset{―}{y}}_{j}-\overset{―}{y}\right)}^{2}, \end{array}$$ (2)

where $\sigma_{n}^{2}=\frac{1}{n}\sum_{i=1}^{n}(y_{i}-\overset{―}{y}{)}^{2}$ is the sample variance of $y_{i}$’s, $y_{i}$ is the outcome of the *i*-th example, and $\overset{―}{y}=\frac{1}{n}\sum_{i=1}^{n}y_{i}$ is the sample mean of $y_{i}$’s. $n_{j}$ is the number of examples in the *j*-th example subset, and ${\overset{―}{y}}_{j}$ is the conditional sample mean of the *j*-th example subset.

Under proper assumptions [12], the predictivity of 𝒮 is lower bounded by


$$\begin{array}{c} \frac{1}{2}+c\times \sqrt{\frac{2I(𝒮)}{n}}, \end{array}$$ (3)

where *c* is an asymptotic constant owing to the assumptions. As suggested by (3), a feature subset with a higher I-score is equivalent to one with a larger value of the lower bound, which means a greater predictivity possessed by this feature subset. This property serves as the theoretical foundation for our proposed algorithm.

I-score quantifies the predictive power of feature subsets by evaluating their interaction with the target outcome. A key requirement of the I-score framework is that all features must be discrete. This is because discretization enhances robustness by reducing sensitivity to small fluctuations in continuous data, allowing the algorithm to identify influential feature interactions more effectively. In this study, we binarize continuous features following the procedure described above. This approach ensures that each feature is split into two meaningful categories, simplifying computations while retaining essential information. Although discretization may introduce some information loss, prior studies have shown that the benefits of robust feature interaction detection outweigh the potential drawbacks. The BDA algorithm then identifies and eliminates dataset-associated features from the discretized feature set, ensuring fairness without significantly compromising classification accuracy. However, we acknowledge that this binary partitioning could oversimplify complex feature distributions. Future research will explore multi-level discretization strategies or alternative I-score formulations that directly handle continuous variables.

It is worth noting that although the I-score and SHAP values [19] both aim to assess feature importance, they serve fundamentally different purposes. SHAP quantifies the marginal contribution of individual features to a model’s predicted output through an additive decomposition framework, typically at the instance level. In contrast, the I-score evaluates the statistical association between feature subsets and the true target variable, emphasizing joint interaction effects rather than marginal contributions to model predictions. Therefore, while SHAP explains how features influence a model’s output, the I-score identifies feature combinations that are strongly associated with the classification target itself. This distinction makes the I-score particularly suitable for detecting dataset-associated signals in our fairness framework.

### 2.2 Backward dropping algorithm

For *p*-dimensional binarized feature vectors, there are $2^{p}$ different subsets of features that require the calculation of the I-score; when *p* is large, it is impractical to perform such computation. For example, when *p* = 512, a typical number of units in the GAP layer of a CNN model, the total number of the calculation of I-scores is 2^512^, which is astronomical. Besides, it is usually the case that a small number of features is sufficient to make a good prediction. The backward dropping algorithm (BDA) was proposed to select such a subset of influential features [12]. BDA is a greedy algorithm. Given an initial feature subset sampled in some manner, the BDA algorithm finds the optimal subset of features by maximizing the I-score through the step-wise elimination of features. We summarize the BDA algorithm we used in Algorithm 1.

In the procedure of our proposed fair I-score method, the BDA algorithm will be repeated by many times to search for more potential subsets of influential features. All the potential subsets of influential features will be ranked by their I-scores, then an I-score threshold will be set via the validation set and the union of those subsets exceeding the threshold will be taken as the final subset of influential features.

**Algorithm 1: Backward Dropping Algorithm (BDA)**

**Input:** validation set of *n* examples of the *p*-dimensional binarized feature vectors, $\left(x_{1},x_{2},...,x_{p}\right)$, and the associated true binary outcome *y*, size of the feature subset *m*

**Output:** 𝒮, the potential subset of influential features

1:  Sample a subset of *m* features such that $𝒮\subset \{x_{1},...,x_{p}\}$

2:  **while** |𝒮|≥1 **do**

3:   Calculate the I-score of 𝒮:

$I(𝒮)=\frac{1}{n\sigma_{n}^{2}}\sum_{j=1}^{2^{\left|𝒮\right|}}n_{j}^{2}{\left({\overset{―}{y}}_{j}-\overset{―}{y}\right)}^{2}.$

4:   Denote $𝒮=\left\{x_{i_{1}},x_{i_{2}},...,x_{i_{\left|𝒮\right|}}\right\}$ with $i_{1}<\cdots <i_{\left|𝒮\right|}$

5:   **for** k=1,2,...,|𝒮| **do**

6:    Tentatively drop the feature $x_{i_{k}}$ in 𝒮 and refer the new subset as ${𝒮}_{k}$

7:    Compute the I-score of ${𝒮}_{k}$, $I({𝒮}_{k})$

8:   **end for**

9:   **if** $\max_{k}I({𝒮}_{k})>I(𝒮)$ **then**

10:    Update $𝒮=S_{k}$

11:   **else**

12:    End the algorithm

13:   **end if**

14:  **end while**

#### Computational considerations.

BDA avoids the infeasible exhaustive search over all $2^{p}$ feature subsets by operating on small candidate subsets of size m≪p. For a given subset 𝒮, the I-score can be computed efficiently by a single pass over the validation set to obtain the required group counts and group-wise outcome means; thus, the per-evaluation cost scales primarily with the number of validation examples *n* (with lightweight bookkeeping over $2^{\left|𝒮\right|}$ binary patterns). Because |𝒮|≤m and we use a small *m* (e.g., *m* = 10, so at most 2^10^ = 1024 patterns), this bookkeeping is inexpensive in practice. Within one BDA run initialized at size *m*, the algorithm evaluates the I-score for the current subset and for each tentative feature drop. In the worst case, the total number of I-score evaluations is bounded by $\sum_{t=1}^{m}(1+t)=m(m+3)/2$, which equals 65 evaluations when *m* = 10. When repeating BDA *R* times (we use *R* = 10,000), the total number of I-score evaluations scales linearly with *R*. Finally, removing the identified features is implemented as a lightweight weight-masking operation (setting the corresponding final-layer weights to zero), which adds minimal overhead and does not require retraining the full CNN. In practice, because *m* is small (e.g., *m* = 10), this selection step is substantially cheaper than training the CNN and is applied only to the validation-set features.

### 2.3 Fair I-score method

**Fig 2:**
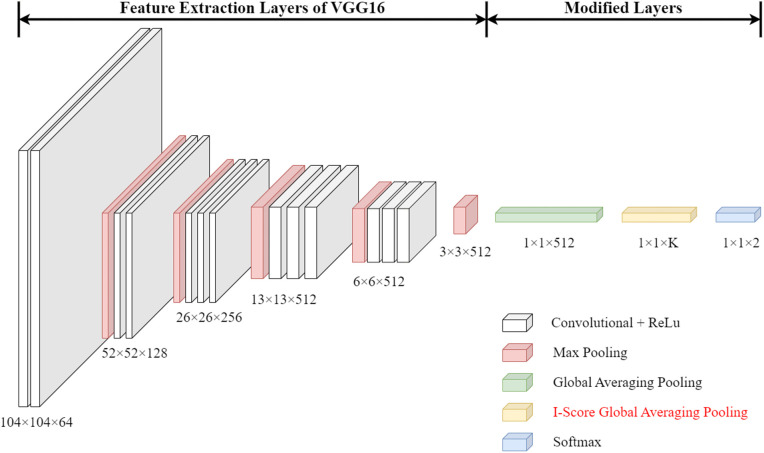
Fair I-score model architecture. This model is built based on the VGG16 architecture. The fully-connected layers in the original VGG16 model are replaced by the global averaging pooling (GAP) layer. The fair features in the global averaging pooling layer will be extracted in the I-score global averaging pooling layer, which is connected to the final classification layer.

In this study, we consider the classification problem of skin lesion images on which we show the effectiveness of our proposed fair I-score method. We use the VGG16 model [5] to demonstrate the proposed fair I-score method through transfer learning. We retain all layers with pre-trained weights before flattening and add a global averaging pooling (GAP) layer that has been shown to provide a better generalization performance in literature [15,20]. The softmax function is adopted as the output layer to produce the predicted class probabilities. The modified model architecture is shown in Fig 2. Moreover, the additional I-score GAP layer is used to select the subset of influential features from the GAP layer after the model training.

**Fig 3:**
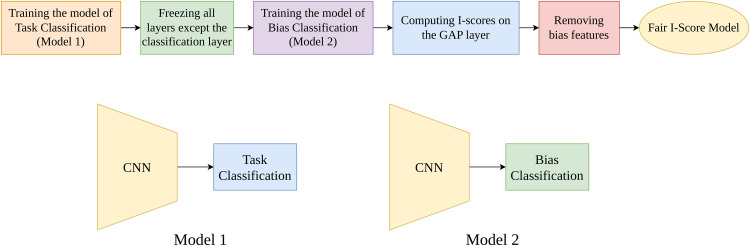
Method framework of the proposed fair I-score method.

The fair I-score method aims to enhance fairness in deep learning models by systematically identifying and eliminating dataset-associated features while preserving predictive performance. The framework of the proposed method, as illustrated in Fig 3, consists of multiple steps, each playing a critical role in mitigating subgroup performance disparities. Below, we provide a detailed breakdown of the methodology.

**Step 1: Constructing the Initial Prediction Model**

The process begins with training a deep learning model (Model 1) for the primary classification task, in this case, skin lesion classification. The model architecture is based on VGG16 with a global average pooling (GAP) layer, which converts feature maps into a vector representation before classification. The GAP layer is chosen because it has been shown to improve model generalization and interpretability. Model 1 is trained on both datasets (ASAN and ISIC) to ensure that it learns representative features from different populations.

**Step 2: Constructing the Dataset-membership Identification Model**

Once Model 1 is trained, we repurpose it to build a secondary model, Model 2, which is designed to capture dataset-associated features. This is done by freezing all layers of Model 1 (except the classification layer) and retraining the output layer with a new objective—predicting the population-specific area (i.e., dataset origin). Since Model 2 now focuses on distinguishing between the ASAN and ISIC datasets, the features it relies on are likely dataset-associated cues (e.g., differences in skin tones, acquisition conditions, or population-/region-related variations). A high accuracy in Model 2 indicates that strong signals predictive of dataset membership exist in the learned representations.

Although geographic origin (dataset membership) is used as the sensitive attribute in this study, the proposed framework is not restricted to a single attribute. When multiple sensitive attributes (e.g., race, gender, or other demographic variables) are of concern, a separate attribute-membership prediction model can be constructed for each attribute using the same procedure as Model 2. The I-score–based feature selection can then be applied individually, and the identified feature subsets may be combined (e.g., via union) to reduce reliance on multiple sensitive factors simultaneously. Therefore, the proposed framework is attribute-agnostic and can be extended to settings involving multiple sensitive attributes.

**Step 3: Extracting Influential Dataset-associated Features Using I-score**

To quantify the importance of individual features in Model 2, we apply the I-score method. The I-score measures the predictive strength of feature subsets by evaluating their interaction with the target variable (in this case, dataset origin). Using the BDA algorithm, we iteratively search for influential feature subsets in the GAP layer of Model 2. The algorithm ranks feature subsets based on their I-score values, ensuring that highly influential dataset-associated features are identified.

**Step 4: Setting I-score Threshold & Selecting Dataset-associated Features**

After obtaining I-score values for multiple feature subsets, a threshold is determined based on the maximum area under the ROC curve (AUC) performance. Features exceeding this threshold are classified as dataset-associated features, as they contribute significantly to distinguishing between population groups rather than the actual classification task.

**Step 5: Constructing the Fair I-score Model**

To mitigate potential unfairness associated with dataset-specific signals, we remove the identified dataset-associated features from the GAP layer of Model 1 while preserving the remaining features. The modified model, referred to as the Fair I-score Model, now operates with features that are not explicitly associated with the dataset origin. This helps ensure that predictions are made without relying on dataset-membership cues (dataset-associated attributes).

It is worth noting that I-score does not directly measure fairness but rather identifies feature subsets that contribute significantly to a classification task. When applied to Model 2 (trained to classify the dataset origin), the I-score helps extract features that are strongly associated with dataset membership (data-source-specific cues). By removing these features from Model 1, we reduce the model’s reliance on dataset-associated signals, thereby improving test-fairness by reducing performance disparity between the two dataset-defined groups.

While this study applies the method to skin lesion classification, the approach is generalizable to other medical imaging domains where fairness concerns are critical. For example, in radiology, models trained on datasets from specific demographic groups may exhibit performance disparities when applied to diverse populations. Similarly, in ophthalmology, AI-based retinal disease diagnosis models may exhibit subgroup performance disparities when training data are imbalanced across populations or acquisition settings. By systematically eliminating dataset-associated features, the fair I-score method can help reduce such disparities and improve the robustness of predictive models across datasets in various medical applications.

*Remark:* To maintain clarity and consistency, we have retained the existing general model names (e.g., Model 1, Model 2, Fair I-score Model) in this work. These names are used conceptually to represent different stages of the proposed methodology rather than specific architectures. This ensures that the framework remains applicable across different neural network architectures without being tied to a single model type.

### 2.4 Evaluation

In this section, the metrics used to evaluate the prediction performance and fairness will be shown in the following. The metrics of the prediction performance include the sensitivity, the specificity, the f1-score, and the ROC curve. To evaluate the model’s fairness, we consider an operational definition called the model fairness indicator motivated by the test-fairness. The ROC curve is used to both evaluate the prediction performance and determine the I-score threshold. Moreover, grad-CAM is adopted to visualize the effectiveness of the proposed I-score method.

#### 2.4.1 Evaluation of model’s performance.

We evaluate different unions of feature subsets according to the order of the I-scores based on Model 2 and the validation set. However, the optimal threshold of the I-score is determined by the maximum value of the area under the ROC curve (AUC) using Model 1.

For each threshold of the I-score, we produce a fair model candidate that is built using the remaining fair features, as determined by the selected I-score threshold. The AUC of the ROC curve of the fair model candidate on the validation set is used to determine the optimal threshold. Then, we evaluate the resulting prediction performance of the selected prediction model on the test dataset; the prediction performance is determined in terms of accuracy, sensitivity, specificity, and f1-score.

Table 1 shows an artificial example that is used to outline the procedure to determine the optimal threshold of the I-score. For example, a threshold I-score value of 1870.51 indicates the top two subsets, Subsets 1 and 2, in Table 1. Then, the union of Subsets 1 and 2 is the feature subset of $x_{403},x_{198},x_{322}$. As an illustrative example, one fair model candidate excludes three identified dataset-associated features while retaining the remaining features for evaluation. This fair model candidate is applied to the validation set to obtain the AUC of the ROC curve. Next, we use the threshold value of 1865.23 to select the union of the top three feature subsets to evaluate the effectiveness of the corresponding fair model candidate with the feature subset of $x_{403},x_{198},x_{322},x_{157},x_{132},x_{99}$. We record all AUC values of the ROC curves for different unions of feature subsets. The optimal threshold of the I-score was determined by the maximum AUC value.

**Table 1:** Subsets of Influential Features with I-scores in Decreasing order.

Subset *t*	Subset of Influential Features	I(𝒮)
Subset 1	{*x*_403_}	1874.23
Subset 2	$\left\{x_{198},x_{322}\right\}$	1870.51
Subset 3	$\left\{x_{157},x_{132},x_{322},x_{99}\right\}$	1865.23
...	...	...
Subset 10000	$\left\{x_{20},x_{90},x_{112},x_{271},x_{511}\right\}$	6.15

#### 2.4.2 Evaluation of model’s fairness.

The definition of test-fairness in (1) provides an idealized criterion for evaluating model fairness. A model would satisfy this criterion if the predicted probabilities are the same across two subgroups defined by a sensitive attribute. However, achieving identical predicted probabilities across subgroups is difficult in practice. Hence, we adopt an operational definition to assess fairness empirically. Consider two subgroups, denoted by sensitive attribute values *a* and *b*. We first compute the sensitivities for subgroup *a* and subgroup *b*, denoted as sensitivity *a* and sensitivity *b*,


$$\begin{array}{c} \text{sensitivity }a=\frac{\text{correct predictions in subgroup group }a}{\text{all data in subgroup group }a}, \end{array}$$ (4)

$$\begin{array}{c} \text{sensitivity }b=\frac{\text{correct predictions in subgroup group }b}{\text{all data in subgroup group }b}. \end{array}$$ (5)

The sensitivity *a* and sensitivity *b* measure the prediction performance for the two subgroups defined by the sensitive attribute values *a* and *b*, respectively. Then, the operational definition of fairness, referred to as the model fairness indicator, is defined as follows,


$$\begin{array}{c} \text{model fairness indicator}=1-|\text{sensitivity }a-\text{sensitivity }b|. \end{array}$$ (6)

The closer the sensitivity *a* and the sensitivity *b*, the higher the value of the model fairness indicator, which indicates the fair I-score model is an effectively fair model.

Intuitively, the model fairness indicator reflects the consistency of model sensitivity across different subgroups. For example, if a model achieves a sensitivity of 0.85 for one subgroup and 0.75 for another, the resulting sensitivity difference of 0.10 indicates unequal detection performance. A reduction of this difference toward zero implies that the model performs more equally across subgroups, which corresponds to improved fairness.

#### 2.4.3 Model visualization.

This study uses grad-CAMs to visualize the test data [14]. After each image is sent to the model trained by the training data, the model outputs the corresponding feature maps and weights of GAP layers selected by the maximum AUC value. We convert the inner product of significant specific feature maps and the value of the GAP layers into heat maps and resized the original images to visualize the effectiveness of the proposed fair I-score method through grad-CAMs.

#### 2.4.4 Fairness definitions and motivation.

In this study, fairness in deep learning models is evaluated using test-fairness, which ensures that the prediction probabilities remain stable across different sensitive attribute groups. Specifically, definitions (4)–(6) in this paper are motivated by well-established fairness principles in machine learning, including equalized odds, demographic parity, and counterfactual fairness.

The notion of equalized odds, introduced by [21], requires that the prediction probability of a positive outcome remains the same for different groups conditioned on the true label. This principle directly aligns with our test-fairness definition, where we ensure that the model does not exhibit disparate performance across dataset-specific sensitive attributes.

An alternative fairness criterion is demographic parity, which requires the predicted outcomes to be independent of sensitive attributes. While demographic parity is widely used in fairness-aware learning, it does not account for true class labels, potentially leading to trade-offs in predictive accuracy. Our approach differs by considering fairness in relation to predicted probability distributions rather than solely focusing on outcome independence.

Another fairness concept closely related to our framework is counterfactual fairness, as proposed by Kusner et al. [22]. This fairness measure evaluates whether a model’s predictions remain unchanged when a sensitive attribute is altered in a hypothetical counterfactual world. Although our test-fairness definition does not explicitly rely on counterfactual analysis, it shares the same fundamental goal of ensuring equitable model performance across different groups.

Beyond theoretical perspectives, fairness has been increasingly studied in medical AI, where dataset heterogeneity and subgroup under-representation can lead to clinically meaningful performance disparities across populations. Recent systematic reviews have summarized a rapidly growing body of fairness evaluation and mitigation work in medical image analysis, highlighting both progress and open challenges in standardizing evaluation protocols and ensuring robust subgroup performance (e.g., [6]). Benchmark efforts further suggest that reported fairness outcomes can be sensitive to dataset choice, model selection, and experimental settings, reinforcing the need for transparent and reproducible fairness evaluation (e.g., [23]). These observations motivate the use of operational fairness metrics, such as the test-fairness definition adopted in this study, to quantify and mitigate performance disparities across dataset-defined sensitive groups.

### 2.5 Comparison with recent fairness-aware approaches

Ensuring fairness in deep learning—particularly in medical image analysis—has become an active and rapidly evolving research area. Recent systematic reviews have summarized a large and growing body of work on fairness evaluation and mitigation in medical imaging, highlighting both methodological diversity and ongoing challenges in achieving equitable performance across demographic and dataset-defined subgroups (e.g., [6]). In parallel, benchmark efforts have emphasized that reported fairness outcomes can vary substantially with dataset choice, backbone architectures, and model-selection criteria, motivating careful and transparent comparisons across methods (e.g., [23]). To contextualize the proposed fair I-score method, we compare it with three widely used categories of fairness-aware deep learning techniques: (i) adversarial debiasing, (ii) reweighting and resampling strategies, and (iii) counterfactual fairness-based methods. In each category, we cite seminal works to define the core idea and emphasize recent medical imaging studies and benchmarks to reflect current progress.

Adversarial debiasing techniques attempt to mitigate bias by introducing an additional adversarial network that predicts sensitive attributes, while the primary model is trained to learn representations that are invariant to these attributes. This approach enforces fairness by discouraging sensitive-attribute information from being encoded in the learned feature space. Foundational work has developed general adversarial debiasing frameworks and adaptive objective balancing for fairness-accuracy trade-offs [24,25]. More recently, adversarial mitigation has been actively explored in healthcare settings, including clinical machine learning pipelines and medical image-related applications, demonstrating practical effectiveness as well as implementation complexity [26]. In dermatology-specific contexts, disentanglement- and contrastive learning-based designs have also been proposed to reduce skin-type information in representations [27]. Although adversarial approaches can reduce bias, they often require additional networks, objectives, and tuning. In contrast, the fair I-score method does not introduce an adversarial component; instead, it explicitly identifies and removes dataset-associated features, making it simpler to integrate and more interpretable.

Another class of fairness techniques focuses on reweighting and resampling strategies, where training instances are assigned different weights or sampling probabilities to improve group balance or enforce fairness constraints. Classic approaches reweight samples to reduce disparate performance across protected groups [28]. In medical imaging, dataset imbalance and subgroup under-representation have been repeatedly shown to yield biased predictive behavior, motivating data-centric balancing strategies as a practical mitigation option [7]. Benchmark studies further compare multiple reweighting/resampling and related mitigation algorithms under unified settings, illustrating both potential gains and sensitivity to experimental choices [23]. While these techniques can be effective, they require modifying the training data distribution and may be constrained by data-access limitations or clinical governance. In contrast, the fair I-score method operates at the model level without altering the original dataset, making it applicable when direct data interventions are impractical.

A third category of fairness-aware techniques relies on counterfactual fairness principles, which require that a model’s prediction remains unchanged if a sensitive attribute were intervened upon while all other relevant factors are held constant. Kusner et al. introduced counterfactual fairness within a causal inference framework using structural causal models [22]. More recent work has advanced practical counterfactual generation and evaluation for high-dimensional data such as images, enabling higher-fidelity counterfactuals and facilitating the analysis of spurious correlations in medical imaging settings [29,30]. While counterfactual approaches provide a strong conceptual grounding, they often require explicit causal or generative modeling and careful validity checks, which can be challenging in complex deep learning pipelines. In contrast, the fair I-score method avoids explicit causal modeling and instead identifies dataset-associated influences via data-driven feature selection, making it easier to integrate into existing architectures.

Overall, recent surveys and benchmarking studies in medical imaging fairness suggest that no single mitigation strategy is universally optimal across datasets, fairness definitions, and deployment contexts, underscoring the need for transparent and interpretable mitigation mechanisms [6,23]. The fair I-score method differs from these existing techniques in several key ways. Unlike adversarial approaches, it does not require additional training objectives or adversarial components, reducing computational complexity. Unlike reweighting and resampling strategies, it does not alter the original dataset, making it applicable to scenarios where dataset modifications are impractical. Unlike counterfactual fairness methods, it does not depend on predefined causal structures, allowing for a more flexible, model-agnostic implementation. Additionally, the use of I-score feature selection provides greater transparency and interpretability, as it explicitly identifies which features are strongly associated with dataset membership (and may contribute to subgroup performance disparities), rather than modifying learned representations in an implicit manner. The proposed method introduces a computationally efficient, interpretable, and model-agnostic approach to fairness improvement in deep learning models. By integrating I-score-based feature selection, it systematically reduces reliance on dataset-associated influences while preserving predictive performance. These advantages position the fair I-score method as a valuable alternative to existing fairness-aware techniques, particularly in applications where explainability and minimal intervention in the data are crucial.

## 3 Experiment results

In this section, we demonstrate the performance of the proposed fair I-score method on the problem of skin lesion classification. The model will be built for classifying two types of skin lesions: nevus and melanoma. In the first experiment, we train two models with different training sets, only the ISIC 2019 dataset, and only the ASAN dataset. The two models trained with only one dataset are used to show that these two datasets can only work well for the test set split from the same dataset. These results indicate a substantial cross-dataset generalization gap between the two data sources, consistent with distributional differences that may contribute to subgroup performance disparity. In the next step, we apply the proposed fair I-score method to reduce reliance on dataset-associated cues and evaluate both prediction performance and test-fairness. Moreover, we adopt a third skin lesion dataset, PAD-UFES-20, to further test external generalization of the fair I-score model.

### 3.1 Data introduction

In this study, the fair I-score method is applied for the skin lesion classification task. Skin cancer is one of the most common cancer in the world. Melanoma, one type of skin lesion, is very fatal and responsible for 75% of skin lesion deaths, though it is the least common skin lesion. It can develop from a common mole or dysplastic nevus on human bodies. The common and primary reason for melanoma is ultraviolet light (UV) exposure in those with low levels of the skin pigment melanin. It shows that the skin color of people or the region people reside is the major risk factor for being diagnosed as the skin lesion. Thus, we consider the region information as the sensitive attribute of the skin lesion in this study.

Most open-source skin lesion datasets do not contain the region information, so we have to adopt several open-source datasets which are collected from different countries to solve this problem. The information on skin lesion data is compiled by [31]. The first dataset is the ISIC 2019 dataset [32–34], which is the major and common skin lesion dataset. The ISIC 2019 dataset was collected from Australia, Austria, Turkey, New Zealand, Sweden, and Argentina; it is referred to as the Western dataset. The second dataset is the ASAN dataset [35], which was majorly collected from South Korea in 2017, and it is referred to as the Eastern dataset. However, the ASAN dataset is a regulated access dataset, so only the downsampling version of the image data can be accessed instead of the original version. The final dataset is the PAD-UFES-20 dataset [36], which was mainly collected from Brazil. The PAD-UFES-20 dataset is only used as an additional test set to evaluate the effectiveness of the proposed fair I-score model. Finally, image data of all three datasets are resized to (104, 104, 3). In this study, both the ISIC and ASAN datasets are divided roughly into training, validation, and test sets at the ratio 8:1:1. The details of the data distributions are shown in Table 2.

**Table 2:** Data Distribution.

Dataset	ASAN		ISIC 2019		PAD-UFES-20	
Area	Eastern		Western		Brazil	
Label^*a*^	NV	MEL	NV	MEL	NV	MEL
Training Set	1,950	432	7,705	3,237	–	–
Validation Set	271	60	1,063	435	–	–
Testing Set	235	59	1,063	434	244	52

^*a*^NV = nevus; MEL = melanoma.

The following subsections expound on how the I-score can be used to improve the fairness behavior in deep learning, with the CNN classification model VGG16 given as an example. For all the model training, we adopt the same hyperparameter setting: the batch size is 32, the optimizer is Adam with the learning rate 10^−5^, the cross-entropy loss function, and the zero-center normalization. The best number of training epochs is determined by monitoring the accuracy of the validation set. All models were trained using a fixed train–validation–test split and a consistent random seed setting to ensure comparability across experiments. Results were not averaged over multiple independent runs; however, this controlled setup enables a fair comparison of the impact of dataset-associated feature removal on subgroup performance disparities. We acknowledge that averaging over multiple random seeds could better characterize stochastic variability in neural network training, and we note this as a limitation.

### 3.2 Fair I-score model for ASAN and ISIC datasets

In order to check whether the different datasets will influence the performance of the models, we first train separately two models with the ASAN and ISIC datasets. The modified VGG16 model without the I-score GAP layer in Fig 2 is used as the model architecture. The model trained with only the ASAN training set is denoted by Model ASAN, and the model trained with only the ISIC training set is referred to as Model ISIC19. The total testing set also consists of the ASAN part (the ASAN testing set) and the ISIC part (the ISIC testing set). The testing result is shown in Table 3. For Model ASAN, the prediction accuracy on the ASAN testing set is 0.956, but the prediction accuracy on the ISIC testing set drops significantly to only 0.765. On the other hand, Model ISIC19 has a prediction accuracy of 0.892 on the ISIC testing set, but its prediction accuracy is only 0.788 on the ASAN testing set. These results indicate a substantial cross-dataset generalization gap between ASAN and ISIC 2019, which may reflect distributional differences between the two data sources. Accordingly, we avoid treating cross-dataset accuracy differences alone as definitive evidence of subgroup bias and quantify unfairness using the test-fairness definition adopted in this study (i.e., subgroup performance disparity under a fixed evaluation setting).

**Table 3:** The Prediction Accuracy for Model ASAN and Model ISIC19.

Model	Testing Set Accuracy	
	ASAN	ISIC
Model ASAN	**0.956**	0.765
Model ISIC19	0.788	**0.892**

To mitigate potential unfairness associated with dataset-specific signals, the fair I-score method is adopted. We first train Model 1 on the combined ASAN and ISIC training sets. Using the same features but labeling each instance by its dataset (ASAN vs. ISIC), we transfer Model 1 to Model 2 to classify dataset membership. Model 2 achieves 0.991 accuracy, indicating strong dataset-discriminative signals in the learned representations. This motivates identifying and removing features that are strongly associated with dataset membership.

Second, we use I-score and the BDA algorithm to extract influential features from the GAP layer in Model 2. There are 512 features in the GAP layer. In this study, the BDA algorithm is repeated 10,000 times, where the initial subsets are randomly sampled with size *m* = 10. The number of examples depends on the validation set and is 271 + 60 + 1,063 + 435 = 1,829. For each repeat of the BDA algorithm, we sample an initial subset and obtain the highest I-score among candidate subsets of features. The median is adopted as the threshold to binarize the feature values. After the I-score threshold is determined by the maximum AUC value, 126 dataset-associated (dataset-discriminative) features are extracted from the GAP layer. Thus, these 126 features are removed from the GAP layer of Model 1, and only the remaining 386 features are used for making predictions. This modified Model 1 is referred to as the fair I-score model. Here, “fair” refers to reduced association with dataset membership under our test-fairness evaluation setting.

#### 3.2.1 Prediction performance of models.

Before evaluating the model’s fairness, we first compare the prediction performance of the fair I-score model and the baseline models (i.e., Model ASAN, Model ISIC19, and Model 1). Table 4 compares the prediction performance of the four models on the ASAN testing set. The fair I-score model achieves the best AUC (0.96). Although other metrics for the fair I-score model are not always the best, it retains strong predictive performance compared with the baseline models. Table 5 compares the results on the ISIC testing set. The fair I-score model achieves the best specificity (0.846), F1-score (0.928), and accuracy (0.896), while the other metrics are comparable to the baseline models. Finally, the comparison on the total testing set consisting of both ASAN and ISIC parts is shown in Table 6. The fair I-score model achieves the best specificity (0.850), F1-score (0.935), accuracy (0.905), and AUC (0.93). Overall, the prediction performance of the fair I-score model is not degraded compared with the baseline models. Fairness is evaluated separately in the next subsection using the test-fairness definition.

**Table 4:** Prediction Performance of 4 Models on the ASAN Testing Set.

Model	Sensitivity	Specificity	F1-Score	Accuracy	AUC
Model ASAN	0.972	**0.881**	0.870	**0.956**	**0.96**
Model ISIC19	0.757	0.796	0.837	0.765	0.87
Model 1	**0.983**	0.847	**0.972**	**0.956**	0.94
Fair I-score Model	0.966	0.879	0.968	0.949	**0.96**

**Table 5:** Prediction Performance of 4 Models on the ISIC Testing Set.

Model	Sensitivity	Specificity	F1-Score	Accuracy	AUC
Model ASAN	0.929	0.442	0.862	0.788	0.80
Model ISIC19	0.923	0.815	0.924	0.892	**0.94**
Model 1	**0.940**	0.776	0.925	0.892	0.91
Fair I-score Model	0.915	**0.846**	**0.928**	**0.896**	0.92

**Table 6:** Prediction Performance of 4 Models on the Total Testing Set Consisting of the ASAN and ISIC Parts.

Model	Sensitivity	Specificity	F1-Score	Accuracy	AUC
Model ASAN	0.937	0.494	0.880	0.816	0.84
Model ISIC19	0.893	0.813	0.910	0.872	**0.93**
Model 1	**0.947**	0.785	0.934	0.903	0.92
Fair I-score Model	0.924	**0.850**	**0.935**	**0.905**	**0.93**

#### 3.2.2 Fairness of models.

Section 3.2.1 has shown that the fair I-score model can retain its prediction performance without using the dataset-associated features, so here we further evaluate the model’s fairness. Here, sensitivity *a* and sensitivity *b* denote the group-wise correct prediction rate (i.e., accuracy) on the ASAN and ISIC testing sets, respectively, as defined in Section 2.4. Table 7 summarizes the results. The model fairness indicator reflects how close *a* and *b* are (a larger value indicates smaller performance disparity between the two dataset-defined groups). The results show that the fair I-score model has the best model fairness indicator (0.947). Specifically, the absolute performance gap decreases from |0.956−0.892|=0.064 (Model 1) to |0.949−0.896|=0.053 (fair I-score model). This indicates that while Model 1 (trained on the combined dataset) already reduces cross-dataset performance disparity compared with single-dataset training, the fair I-score model further improves test-fairness by removing features that are strongly associated with dataset membership.

**Table 7:** Evaluation of Model’s Fairness.

Model	Sensitivity *a*	Sensitivity *b*	Model Fairness Indicator
Model ASAN	0.956	0.788	0.832
Model ISIC19	0.765	0.892	0.873
Model 1	0.956	0.892	0.936
Fair I-score Model	0.949	0.896	**0.947**

#### 3.2.3 Discussion.

The BDA algorithm systematically removes dataset-associated features identified via the I-score ranking process to improve fairness while preserving classification performance. However, an important consideration is whether the removal of features negatively impacts model accuracy.

To evaluate this, we compared the performance of the Fair I-score Model against the baseline models (Model ASAN, Model ISIC19, and Model 1) across multiple metrics, as shown in Tables 4–6. The results indicate that accuracy, sensitivity, and specificity remain stable, suggesting that the removal of dataset-associated features does not significantly degrade classification ability. This is because BDA preserves essential diagnostic features while selectively eliminating features strongly associated with dataset membership (i.e., data-source-specific cues).

Furthermore, the fairness metric, represented by the model fairness indicator, shows clear improvement in the Fair I-score Model compared to Model 1. This demonstrates that removing dataset-associated features helps balance prediction performance across different dataset groups without sacrificing overall effectiveness. These results confirm that Algorithm 1 effectively enhances fairness while maintaining high classification performance, making it a practical solution for mitigate subgroup performance disparities in deep learning models.

#### 3.2.4 Model visualization.

**Fig 4:**
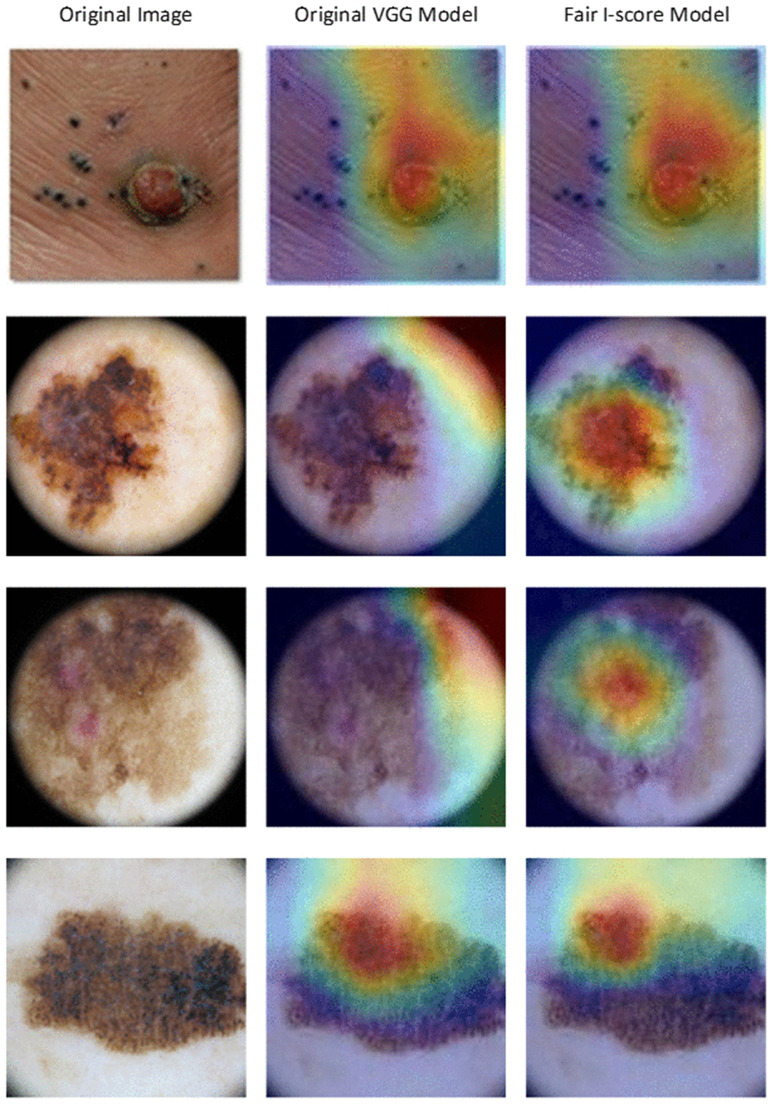
Grad-CAM visualizations of randomly selected testing images. The left column shows the original images, the center column shows the Grad-CAM results of the original VGG16 model (Model 1), and the right column shows the Grad-CAM results of the fair I-score model. Warmer colors indicate regions that contribute more to the predicted class. Across representative examples, Model 1 sometimes assigns high importance to dataset-associated background cues (e.g., peripheral image borders, illumination gradients, or non-lesion regions), whereas the fair I-score model shifts attention toward lesion regions. This qualitative comparison illustrates that removing dataset-associated features can reduce reliance on background cues while preserving diagnostically relevant attention patterns.

In Fig 4 we visualize the original VGG16 model, Model 1, and the fair I-score model by Grad-CAMs. For the results of the second and third rows, instead of the skin lesion parts, Model 1 makes predictions based on background cues that are strongly associated with dataset membership (data source). In contrast, the fair I-score model attends more to the lesion regions when making predictions. In addition, the results in the first and last rows show that the fair I-score model can focus more on the lesion parts.

### 3.3 Independent test on the PAD-UFES-20 dataset

As an independent test, we adopt the third dataset, PAD-UFES-20, which is collected from Brazil. Table 8 summarizes the results on PAD-UFES-20 for Model 1 and the fair I-score model. Because PAD-UFES-20 does not provide sensitive-group annotations compatible with our test-fairness definition, a subgroup fairness score cannot be computed for this external test set. As an external validation of robustness and generalization, the fair I-score model achieves comparable accuracy and a higher AUC than Model 1 on PAD-UFES-20.

**Table 8:** Prediction Performance of Model 1 and Fair I-score Model on the PAD-UFES-20 Testing Set.

Model	Accuracy	AUC
Model 1	0.811	0.68
Fair I-score Model	**0.814**	**0.71**

## 4 Discussion and conclusion

The techniques of deep learning have become more and more mature for solving various application problems, but there are more issues raised to be addressed. The fairness of the model is one of these important issues. If the training data contain sensitive information about specific ethnic groups or populations, the model may learn spurious group-associated cues, which can lead to subgroup performance disparities across different groups. In this study, we propose the fair I-score method to deal with the problem. On top of the GAP layer of the CNN model, the fair I-score method involves the feature selection mechanism that calculates the influence of a feature subset by I-score and extracts the dataset-associated features by the backward dropping algorithm. By eliminating the dataset-associated features and retaining the fair features through thresholding, the model can keep its prediction performance and increase its fairness at the same time. We adopt the fair I-score method on a VGG16 model and use the problem of skin lesion classification to demonstrate the proposed method. The result shows that I-score can be used to effectively select influential features in the GAP layer of the VGG16 model. And the resulting fair I-score model can reduce reliance on dataset-associated influences while retaining predictive performance.

The effectiveness of the proposed fair I-score method was evaluated in this study under an operational definition of fairness involving a single sensitive attribute with two categories. Although dataset membership (geographic origin) was used as the sensitive attribute in our experimental setting, the framework itself is not restricted to binary attributes. In practice, multiple sensitive attributes may be relevant to the task of interest, and each attribute may contain more than two categories. As discussed in Section 2.3, the attribute-membership modeling step can be applied separately to different sensitive attributes, and the resulting feature subsets can be combined to reduce reliance on multiple sensitive factors. Future extensions may further investigate more complex scenarios, such as multiclass classification problems and alternative model architectures beyond the GAP structure and CNN models, including recurrent neural networks and transformers.

It is worth noting that the proposed fair I-score method is decoupled from the mode training of the task of interest. After the original model is built, the proposed fair I-score method searches for dataset- or group-associated features that contribute to subgroup performance disparities from a fixed set of features and removes them to improve model fairness while retaining predictive performance. We are developing approaches that incorporate the concept of I-score into the model training so that the resulting model has a direct and better trade-off between fairness and prediction performance. After all, a fair model with poor prediction capability is useless in practice. Furthermore, we may also incorporate different definitions of fairness into the model training such as counterfactual fairness that addresses causality [37].

While this study focuses on skin lesion classification, the proposed fair I-score method has the potential to be applied across various medical imaging tasks where fairness concerns arise. In fields such as radiology, ophthalmology, and pathology, disparities in training data can lead to biased model predictions, disproportionately affecting underrepresented populations. For instance, in chest X-ray classification, models trained predominantly on datasets from certain demographic groups may perform poorly on others, similar to the bias observed in dermatology datasets. By identifying and removing dataset-associated features, our method could enhance fairness in deep learning models used for disease detection and diagnosis. Future work will explore the application of the fair I-score method in additional medical imaging contexts, assessing its effectiveness in mitigate subgroup performance disparities in broader healthcare AI applications.

This study demonstrates the fair I-score method using VGG16, but the approach is not limited to this architecture. The methodology is designed to be architecture-agnostic, meaning it can be applied to other CNN-based models and even extended to non-CNN architectures, such as transformers in medical imaging. Future work will involve testing the fair I-score method across diverse architectures, including ResNets, EfficientNets, and Vision Transformers. By validating the fair I-score method on these architectures, we aim to demonstrate its broad applicability across different neural network frameworks, ensuring fairness improvements in various deep learning settings.

While the fair I-score method effectively reduces subgroup performance disparities in deep learning models, several limitations should be acknowledged. First, the method relies on an initial dataset-membership model (Model 2) to extract dataset-associated features, meaning its effectiveness depends on the extent to which dataset-membership–predictive signals are present in the data. Second, the approach is currently applied to binary classification tasks, and its generalization to multi-class medical diagnoses requires further exploration. Third, the computational overhead of I-score feature selection may be a concern for large-scale medical imaging datasets, necessitating optimization for efficiency. Finally, fairness evaluation in medical AI is complex, and incorporating causal fairness techniques (e.g., counterfactual fairness) could further enhance model robustness. Consistent with recent reviews and benchmarks (e.g., [6,23]), standardized and deployment-aware fairness evaluation remains an important direction in medical imaging. Future research will focus on extending the fair I-score method to multi-class and multi-modal medical imaging tasks, integrating real-world clinical data, and exploring fairness definitions tailored to healthcare applications to ensure equitable AI-driven diagnostics.
